# The Beighton Score as a measure of generalised joint hypermobility

**DOI:** 10.1007/s00296-021-04832-4

**Published:** 2021-03-18

**Authors:** Sabeeha Malek, Emma J. Reinhold, Gemma S. Pearce

**Affiliations:** 1 Centre for Mechanochemical Cell Biology, Division of Biomedical Sciences, Warwick Medical School, University of Warwick, Coventry, CV4 7AL UK; 2 Royal College of General Practitioners, London, UK; 3 Faculty of Health and Life Sciences, School of Psychological, Social and Behavioural Sciences, Coventry University, Coventry, CV1 5FB UK

**Keywords:** Beighton score, Generalised joint hypermobility, Joint hypermobility, Joint hypermobility syndrome, Ehlers–Danlos Syndrome, Range of motion, articular

## Abstract

The Beighton Score (BS) is a set of manoeuvres in a nine-point scoring system, used as the standard method of assessment for Generalised Joint Hypermobility (GJH). It was originally developed as an epidemiological tool used in screening large populations for GJH, but later adopted as a clinical tool for diagnostic purposes. Its ability to truly reflect GJH remains controversial, as joints within the scoring system are predominantly of the upper limb and disregard many of the major joints, preventing a direct identification of GJH. Furthermore, a consistent finding in the literature whereby the BS failed to identify hypermobility in joints outside the scoring system suggests its use as an indirect indicator of GJH is also not viable. As such, the collective findings of this review demonstrate a need for a change in clinical thinking. The BS should not be used as the principle tool to differentiate between localised and generalised hypermobility, nor used alone to exclude the presence of GJH. Greater emphasis should be placed on a clinician’s judgement to identify or exclude GJH, according to its full definition.

## Introduction

Joint Hypermobility (JH) is not a diagnosis, but a descriptor, used to define a joint that exceeds its normal Range of Motion (ROM), taking into account age, sex and race [1, 2]. This feature is predominantly determined by the tightness or laxity of ligaments, which in turn, is influenced by genetics, involving the connective tissue genes collagen, elastin, and fibrillin [2]. As a consequence, Heritable Connective Tissue Disorders (HCTD) like the Ehlers–Danlos Syndromes (EDS), Marfan Syndrome, and Osteogenesis Imperfecta, result in systemic ligamentous laxity and can present with Generalised Joint Hypermobility (GJH). The diagnosed prevalence of the conditions EDS and Joint Hypermobility Syndrome (JHS), which are associated with GJH, has been found to be 1 in 500 [3], although GJH is also present in the general healthy population. GJH is also a descriptor, and is defined as the simultaneous presence of JH at the four limbs and axial skeleton, with involvement of both the major and minor joints [1].

The BS has been used as the standard method of assessment of GJH in research studies, as well as in all present and previous diagnostic criteria for the hypermobility syndromes. The BS has its origins in 1964 when Carter and Wilkinson devised a scoring system to define GJH to investigate its association with congenital hip dislocation [4]. GJH was considered positive if the individual scored 3 or more from five criteria, requiring both upper and lower limbs to be involved from the following: apposition of the thumb to the forearm, dorsiflexion of the ankle, as well as hyperextension of the elbows, knees, and all the metacarpophalangeal joints (MCPJs). This method was later modified by Beighton et al. to determine the epidemiology of GJH in an African population [5]. Hyperextension of all the MCPJs was replaced with just the little finger beyond 90°, and dorsiflexion of the ankle was replaced with forward flexion of the trunk, creating the BS out of a total of 9 which is widely used today.

Alternative scoring systems to the BS exist. This includes the Rotès–Quérol scoring system, which includes additional measurements of the cervical and lumbar spine, shoulder, hip, and metatarsophalangeal joints (MTPJs) to give a total score of 11 [6]. The Hospital Del Mar criteria have a score of 10, and include the thumb, MCPJs, MTPJs, elbows, shoulders, hip, knee, patella, ankle/feet, and an assessment for ecchymoses or easy bruising [6]. Since both these scoring systems are more time consuming in nature, they have consequently seen limited use in practice, while the BS has become the standard method of assessment.

Since its creation in 1973, the BS has remained unchanged, and adopted both for research purposes and as a clinical diagnostic tool. However, it was originally developed as an epidemiological tool, involved in screening large populations for GJH. Neither Carter and Wilkinson [4] nor Beighton et al. [5] provide any evidence-based justification for the selection of joints within the assessment method. It appears joints were not specifically selected to accurately reflect GJH or hypermobility present in other joints, but chosen instead on a functional basis, for ease of access and efficiency without the need for equipment. As a result, two thirds of the joints being assessed are located in the upper limbs and many of the major joints are disregarded, and with only a single plane of joint motion measured. In addition, the method is an ‘all or nothing’ system, only determining the presence of hypermobility and giving no indication of its severity. There is no clear description or guidance in the original text for how the test should be performed, or whether the active or passive ROM for some of the joints should be measured. While GJH is generally indicated by a score of ≥ 4/9 in adults, we can find no evidence-based justification for use of this cut-off value. Its ability to truly reflect GJH is, therefore, questionable and this is concerning. From a nosologic perspective, it could lead to the incorrect classification of disease, which in turn has implications for research. There are potential consequences for the development of valid molecular diagnostic techniques, as well as effective treatment and management strategies for patients. There are further concerns for its use in clinical practice and the socioeconomic impact this may have. Unrecognised hypermobility disorders may lead to patients being denied access to the appropriate healthcare services, as well as the necessary disability support needed to enable employment, increase economic output and lead a fulfilling social and family life. Indeed, this is a difficulty already described by those with a diagnosis of EDS [7]. The continued use of the BS as a clinical diagnostic tool, particularly within the 2017 International Classification of EDS for the diagnosis of hypermobile EDS (hEDS) [8], therefore, remains controversial since it was originally intended as a screening tool. Despite this, no thorough examination or review of its clinical properties has yet been performed. The aim of this paper is to review the validity and reliability of the BS as an assessment method to classify GJH, and further discuss its suitability for diagnostic purposes.

##  Methods

A narrative literature search formed the basis of this review. A formal meta-analysis was not attempted due to substantial heterogeneity in study methodology, GJH classifications and BS cut-off points. Instead, the aim of the literature search was to identify and present all relevant studies assessing the clinical aspects of the BS to enable a discussion regarding its use as a diagnostic tool, and more specifically, its ability to exclude the presence of GJH.

The electronic databases of PubMed and Scopus were chosen and a Boolean search strategy was employed to identify relevant articles published in the English language before Oct 2020. Studies were initially identified through use of the search term “Beighton Score”, alongside “validity”, “correlation”, or “reliability”. The search was expanded for validity by including various joints as search terms, such as “shoulder”, “temporomandibular joint”, “ankle”, etc., and further papers were identified through a snowballing approach.

Eligibility criteria for validity included specific study design and participant demographic. Studies were required to examine a statistical association between the BS and a measurement of hypermobility in other joint(s) in participants reflective of a representative population. Studies in which participants were exclusively from hypermobile skewed populations such as children and gymnasts were excluded, unless findings were of significant relevance to the discussion of the research topic.

## Validity of the Beighton Score

There is no gold standard test for classifying GJH in an individual. However, the BS itself is now often considered and used as such, though as stated, this was not its original purpose. Neither Carter and Wilkinson [4] nor Beighton et al. [5] devised the BS for diagnostic purposes, and as such, it does not appear to have been validated by examining its association with other hypermobile joints and its ability to truly detect widespread GJH in adults.

The BS has, however, been validated in children [9]. Here, in 500 children aged 6–12 years, 16 ROMs in eight different joints were measured using the extended standardised joint mobility protocol and compared with the BS. Those children classed as hypermobile (BS ≥ 5) were found to have a significantly increased ROM in all other joints measured, including the ankle, hips, and shoulders, as well as an association with other features indicative of a hypermobility syndrome, like pes planus [9]. This study demonstrates that the BS is a valid method for determining GJH in children. However, hypermobility is known to be highly prevalent in children and also diminishes with age [5, 10]. As such, children with a positive BS are more likely to present with hypermobile joints outside the BS, and so the same inferences on its validity cannot be made with regards to its use in adults. Though no study has directly validated the BS for GJH in adults, this can be inferred by examining its association with hypermobility present in other singular joints.

It has been demonstrated that the BS does not correlate with hypermobility of the shoulders [11, 12]. In one study, the BS and various measures of shoulder laxity were taken from 160 individuals aged 16–35 years, and no correlation was found between the measurements, with a positive BS (≥ 4) showing low sensitivity and low positive predictive values for shoulder laxity [12]. Even when the BS cut-off value was raised to ≥ 6, there was no significant increase in the positive predictive value [12]. In addition, the BS may not necessarily reflect joint instability, as demonstrated in another study which found no relationship between a BS of ≥ 6 and instability of the shoulder [13]. The shoulder is of particular clinical relevance as it is often reported by hEDS patients to be the most troublesome [14] and most prone to dislocation [15, 16]. Some have suggested that shoulder dislocation may even be the first presenting sign of hEDS [17]. Indeed, in a study of over 100 patients with a strong suspicion of EDS based on a presentation of symptoms, family history and other physical findings, 45 had a negative BS (BS < 4). However, their mean glenohumeral abduction was still 20° higher than normal, highlighting the significance of shoulder hypermobility, even in potential patients who may have a negative BS [18].

A lack of correlation has also been found between the BS and laxity of the joints in the lower limb. In one study, the BS and instrumented measurements of knee and ankle laxity were taken from over 50 individuals with a mean age of 21, and a positive BS (≥ 4) showed non-significant correlations with those measurements, with the authors concluding that both knee and ankle laxity are joint specific and not generalisable [19]. Another in over 140 children aged between 13 and 15 years found no correlation between the BS and ankle dorsiflexion [20]. However, some studies contradict these findings. A smaller study in over 30 adults found a weak but significant association between the BS and instrumented measurement of knee laxity as well as knee instability [21], while another found those with a BS ≥ 6 had a statistically significantly higher ankle dorsiflexion range by 4° [22], though this value may not be clinically significant.

The BS has been found to correlate with spinal mobility [23, 24]. In a study of over 60 individuals, those with a BS of ≥ 4 demonstrated significantly increased spinal intervertebral mobility, though this was assessed through functional radiographs and not through physical manoeuvres [24]. The manoeuvre presumed to measure spinal hypermobility within the BS is the forward flexion of the trunk manoeuvre. However, this does not appear to truly reflect inherent hypermobility of the spine or axial skeleton, as the manoeuvre is known to be trainable, as demonstrated by ballet dancers [25], and is also known to be affected by hamstring length [26]. A study in men found shorter hamstring lengths to be associated with a decreased ROM of both the pelvic and lumbar angle, restricting the forward flexion manoeuvre [26]. Of clinical significance is the contribution of muscle retractions on this movement, particularly in hypermobile patients. In one study of over 200 hypermobile patients (BS ≥ 5) aged 2–70 years, 87.5% were found to have muscle retractions which prevented them from performing the manoeuvre [27]. From this study, the authors demonstrated that 84.2% of hypermobile patients, presenting with a current BS of ≥ 5, were unable to perform the forward flexion manoeuvre which would give them one further point on the BS [27]. It is possible, therefore, that many people scoring below the current diagnostic cut-off values may be deprived of a diagnosis due to the presence of such muscle retractions, again raising concerns regarding the validity of the BS for diagnostic purposes.

The same finding has also been noted in hypermobile children, even before age-related loss of hypermobility is expected. In a study of over 400 children between 6 and 11 years of age, 86% of those who were hypermobile (BS ≥ 5) could not perform the manoeuvre [28], while another in over 200 children aged 10–13 years, found 84% of hypermobile males (BS ≥ 4) and 78% of hypermobile females (BS ≥ 5) could not perform the forward flexion manoeuvre [29]. When compared with children without GJH, no differences were found between the groups in their abilities, and further lack of differences was found in the flexibility of the trunk and the muscle–hip complex [29]. It is thought that hypermobility leads to alterations in the activation of the pelvic and lower limb musculature to compensate for joint instability [30], restricting lumbar movement and preventing forward flexion. It has been suggested that the presence of muscle retractions and an inability to perform the manoeuvre may actually be indicative of hypermobility [27]. One study has concluded that while this manoeuvre has high specificity (93.7%), its sensitivity is so low (13.8%) that it adds no additional value to the BS [28]. These findings, in which the vast majority of confirmed patients are unable to perform the very manoeuvre contributing to their diagnosis, should call into question its continued inclusion in the BS for diagnostic purposes.

Another joint of particular clinical relevance is the Temporomandibular Joint (TMJ), and it is becoming increasingly recognised that there is a relationship between both GJH and hEDS, and TMJ Disorders (TMD) [31]. TMD is characterised by a symptomatic presentation of pain or discomfort associated with the TMJ, with a decreased functionality in opening and chewing motions. Most studies examining this relationship have found the prevalence of GJH to be higher in the TMD population than normal controls [31]. However, this relationship has not necessarily translated into direct correlations between GJH (BS ≥ 4) and hypermobility of the TMJ itself. A study in 60 TMD patients found no significant correlation between a positive BS and TMJ hypermobility as measured by lateral X-rays [32], while another in over 40 female volunteers found that the range of mandibular motion did not significantly differ between those with a positive and negative BS [33]. This is further supported by two studies in over 60 female TMD patients, where the BS did not correlate with MRI-evident displacement of the TMJ [34, 35]. However, a conflicting study found a significant positive correlation between mandibular ROM and a positive BS in a study of over 30 women with TMD [36]. Another found a weak but significant positive association in over 200 15–16-year-old hypermobile girls [37]. It is plausible that those with GJH may initially present with TMJ hypermobility, but repeated trauma including subluxations and dislocations facilitates the development of TMD, resulting in limited mobility of the TMJ itself. These studies demonstrate that in those who develop a symptomatic presentation, joint ROM itself may not always be the most reliable indicator of inherent ligamentous laxity.

The BS appears to correlate better with joints of the upper limbs, including the thumb and wrist. A study in over 160 individuals found that the BS correlated moderately and significantly with laxity of the thumb when measured via stress view radiograph [38], while a study in 50 women found a low but significant correlation between BS and assessments of wrist laxity [39].

## Reliability of the Beighton Score

The reliability of the BS simply refers to its ability to produce consistent results. More specifically, intra- and inter-examiner reliability refers to the same or different examiners, respectively, in their ability to interpret and allocate the same BS to the same individual. This is of note, as the BS is an “all or nothing” system. It does not measure the degree of hypermobility in each joint, only assigning a positive score if the joint ROM passes the required threshold. Therefore, joints presenting with borderline hypermobility are left open to interpretation on its scoring by different examiners or on different occasions.

This is particularly relevant as various circumstantial factors may promote or diminish inherent joint ROM, and influence the overall BS. For example, various studies have shown that hypermobility is diminished on the dominant side of the body [5, 40, 41]. Stretching and warming up have been shown to increase joint ROM [42]; while temperature, both heat and cold, have been shown to affect the flexibility of tendons and ligaments, ultimately influencing joint ROM [43, 44]. In addition, hormonal fluctuations during the menstrual cycle are thought to affect laxity of the knees [45]. Together, it is conceivable that an individual’s BS could be dependent on circumstantial factors at the time of examination, such as climate, temperature, stage of menstrual cycle, and prior physical activity, particularly in those with a borderline presentation. In addition, there is no agreed consensus on how the test should be performed or interpreted. For example, whether the passive or active ROM of the joints should be measured, whether historical hypermobility should also be considered, or whether any allowances for exceptions such as injuries, surgery, or even pregnancy should be made. These factors could further impact its reliability when performed by different examiners or on different occasions. Therefore, the ability of the BS to consistently and reliably assign an individual with GJH is relevant, particularly for clinical applications.

Reliability has been examined in various studies at the level of both individual manoeuvres within the BS, for example, agreement on assigning a positive score for a particular joint, but also the overall score and classification of GJH, for example, agreement on classifying an individual with a BS of 5, and therefore with GJH. The kappa statistic is the most often used correlation statistic used to analyse reliability, which calculates the percentage agreement between two scores while also taking into account chance agreement, i.e. the possibility that examiners may correctly guess the scoring in the event of uncertainty rather than providing a genuine interpretation, and thereby providing a more accurate representation of reliability [46]. The kappa statistic ranges from − 1.0 to + 1.0, with negative values indicating disagreement, zero indicating no agreement, and a value of 1.0 indicating perfect agreement. Generally, most reliability studies have consistently produced kappa values between 0.4 and 0.8, indicating the BS to demonstrate moderate intra- and inter-examiner reliability in both adults and children (Table 1) [6, 47–52].

**Table 1:** Intra- and inter-examiner reliability for positive hypermobility findings for individual manoeuvres, GJH classification, and agreements with overall score

	Intra-examiner reliability—adults	Inter-examiner reliability	
		Adults	Children
Individual manoeuvres			
Hyperextension of the fifth finger—Kappa	0.78 [47] 1.00 [47]	0.40 [48] 0.61 [48] 0.70 [47] 0.73 [47] 0.79 [6]	0.43 [49] 0.48 [49] 0.49 [49] 0.69 [49]
Apposition of the thumb to the forearm—Kappa	0.52 [47] 0.71 [47]	0.87 [6] 0.91 [47] 0.94 [48] 1.00 [47] 1.00 [48]	0.82 [50] 0.82 [49] 0.84 [49] 0.89 [49] 0.94 [49]
Hyperextension of the elbows—Kappa	0.61 [47] 0.76 [47]	0.21 [47] 0.34 [48] 0.50 [47] 0.64 [48] 0.92 [6]	0.68 [50] 0.54 [49] 0.60 [49] 0.63 [49] 0.73 [49]
Hyperextension of the knees—Kappa	0.19 [47] 0.84 [47]	0.31 [47] 0.40 [47] 0.85 [48] 0.86 [6] 0.90 [48]	0.30 [49] 0.43 [49] 0.44 [50] 0.62 [49] 0.62 [49]
Forward flexion of the trunk—Kappa	1.00 [47]	0.88 [48] 0.93 [6] 0.94 [47]	0.64 [49] 0.82 [50] 0.84 [49]
Classification of GJH (BS ≥ 4)—Kappa	0.78 [47]	0.54 [47] 0.78 [48]	–
Classification of GJH (BS ≥ 5)—Kappa	0.47 [47]	0.66 [47] 0.66 [48]	0.59 [49] 0.64 [49]
Agreement with overall score—Spearman’s $\gamma$	0.86 [51]	0.87 [51]	
Agreement with overall score—Intraclass Correlation Coefficient (ICC)	0.76 [47] 0.84 [52] 0.92 [52]	0.72 [47] 0.89 [52] 0.94 [52]	

However, as a research and clinical diagnostic tool, moderate reliability may not be sufficient. Kappa values below 0.6 have been suggested to be inadequate and, therefore, potentially not appropriate in a health care or clinical research setting [46], yet this is a value which is not consistently met in the aforementioned studies (Table 1). In addition, a recent systematic review performed a best-evidence synthesis for the reliability of the BS, using the Consensus-based Standards for selection of health Measurement Instrument (COSMIN) checklist [53]. This checklist evaluates the methodological quality of the reliability studies included in the systematic review, which are then analysed alongside the results of the actual studies, while taking into account the number of studies included and the total sample size. From the 5 criteria—strong, moderate, limited, conflicting, and unknown—the review rated the overall strength of evidence to support the reliability of a positive BS to be limited to conflicting [53]. While this was the best performing GJH assessment method from those included, the review still demonstrated that there is not sufficient evidence to entirely support the use of the BS as a diagnostic tool. While the BS has not demonstrated poor reliability, more research is needed to clarify its suitability for clinical and research purposes. Better standardisation of the BS with an agreed consensus and clearer guidelines produced as to how it should be performed and interpreted could improve reliability, particularly for clinical applications.

## The Beighton Score cut-off points: the influence of age, sex and race

The original study by Beighton et al. appears to have classified GJH arbitrarily, requiring a BS cut-off of ≥ 4 [5], which is the definition generally used in most studies. Adjustments to the cut-off value, however, are often debated, to take into consideration factors that influence GJH such as age, sex, and race [9, 10, 54–56]. The influence of these factors have been demonstrated in studies examining the prevalence of hypermobility within and between these populations, as well as their relationship with symptoms.

With regards to racial differences, several studies have shown a general trend of Caucasian populations demonstrating a lower prevalence of GJH [10, 57–59], which is increased in Asian [59–61], African [59, 62], and Arab [40, 59] populations. However, it has not yet been established if this is associated with a higher prevalence of hypermobility-related symptoms, with studies demonstrating conflicting results. A study in West Africans showed no increase in articular symptoms [62]. However, one study with Indian schoolchildren participants showed an association between higher BS and musculoskeletal pain [63], while another study with Iraqi University student participants demonstrated a significant correlation with both articular symptoms and syndromic features [40].

The prevalence of hypermobility has consistently been shown to be higher in females across the lifespan [5, 10, 40, 41, 54, 56–59, 62, 64–68]. These differences become more pronounced around the age of 14, with females demonstrating significantly higher rates of GJH [10, 54, 56], while the decrease in hypermobility following adolescence is more pronounced in males [69].

Prevalence rates of hypermobility within specific age groups are difficult to accurately discern due to sample ages and sex varying considerably between and within studies. However, hypermobility has consistently been shown to be highly prevalent in the youngest children and shown to decrease with age, falling rapidly throughout childhood and then at a slower rate during adulthood [5, 10, 60]. This decreasing trend is demonstrated in various studies in children and adolescents, with the prevalence of GJH (BS ≥ 4) ranging from 64.6% in children aged 4–7 [65], 35.6% at age 10 [70], 9.4% in those aged 12–13 years [64], and 11.7% in children aged 13–19 [68].

While the prevalence of hypermobility is significantly higher in younger children, some studies have shown that this is not associated with increased musculoskeletal symptoms [66, 71], though there is other evidence which contradicts this [63]. From over 380 children examined at ages 10–12, those with a BS of ≥ 6 were shown to be more likely to suffer musculoskeletal pain at a 4-year follow-up, and that this was an independent predictor for pain recurrence [72]. Additionally, in children presenting with lower limb pain, those who were hypermobile (BS ≥ 6), were also found to have a threefold significantly increased risk for lower limb pain recurrence after 4 years [73]. However, one study has shown that within the hypermobile population, symptomatic children can be differentiated from asymptomatic children through other findings [74]. These symptomatic children demonstrated significantly higher skin extensibility, and the degree of hypermobility in each joint was also higher. There was also a significant increase in collagen degradation products like hydroxyproline in the urine, alongside significantly decreased ultrasound measurements in the bone indicating lower bone density, as well as a lower diastolic blood pressure [74].

Hypermobility continues to decline throughout adulthood, although at a slower rate than is seen in childhood [5, 10, 60], with the probability of being classified with GJH (BS ≥ 4) decreasing 5.5% for every 1 year increase in age [10]. In a study of 200 individuals over 70 years of age, no one was found to have a BS higher than 2 [10]. The loss of hypermobility with age has also been specifically demonstrated within the hypermobile population, with the delineation of three distinct phases [75–77]. The first decade of life, i.e. the “hypermobility” phase, presents with marked hypermobility, with joint sprains and strains occurring in around 40% of patients. The second “pain” phase occurs in the second decade of life and is characterised by widespread chronic pain with increased joint instability and decreasing hypermobility. Finally, the third “stiffness” phase is characterised by a dramatic decrease in JH with a diminished quality of life. A cut-off age of 33 has been established, after which most diagnosed hypermobile patients will not reach the cut-off of 4 on the BS [76], questioning its suitability in the diagnosis of adult patients.

## Discussion

Any clinical test or method used for diagnostic purposes must be a valid and accurate indicator for disease. For GJH, this would be a method that accurately identifies JH at both major and minor joints of the four limbs and axial skeleton. The efficacy of such methods is formally determined by comparisons to the gold standard method through measurements of sensitivity and specificity. However, no official gold standard method exists for GJH to allow such measurements of the BS. Its sensitivity and specificity, however, can be inferred by examining its ability to identify those whose hypermobility meets the definition of GJH.

With regards to sensitivity, this is an aspect of the test that allows identification of all those with the disease. For this to be clinically useful, a negative result should effectively exclude the presence of disease. However, this review has raised concerns about the ability of the BS to do this. As described, the selection of joints within the scoring system does not accurately represent the definition of GJH and, therefore, cannot be used as a direct indicator of GJH. Neither can the BS value be used as an indirect indicator of GJH, as several studies have demonstrated no association of the BS value with hypermobility present in other joints. Therefore, a positive BS value is unable to effectively identify all presentations of GJH and false-negative outcomes become feasible. Here, individuals may receive a negative BS outcome, yet may still present with JH in locations outside the scoring system that fulfil the definition of a GJH. The sensitivity of the BS, therefore, is not sufficient to exclude GJH in individuals. This also relates to specificity.

Specificity refers to the ability to effectively identify all those without the disease. This aspect of the BS would be clinically useful only if a positive result could effectively confirm the presence of GJH. However, the selection of joints within the BS prevents its use as a direct positive indicator of GJH, as this value bears no reflection on the location, spread, and type of joints that have been affected by hypermobility. Therefore, the possibility of false-positive outcomes is also feasible. For example, an individual with localised hypermobility limited to the upper limbs could potentially generate a BS of up to 6 and an incorrect classification of GJH, yet this same value can also be reached by an individual with a genuine presentation of GJH. Therefore, the BS does not appear to be a sufficiently specific tool to differentiate between generalised or localised hypermobility and enable an effective delineation of GJH. Indeed, the need to differentiate between the two is of clinical importance, with a generalised presentation indicating the presence of systemic ligamentous laxity and, hence, a potential HCTD. The inability of the BS to effectively do this is significant for its use as a clinical and diagnostic tool.

There is widespread debate in the literature about adjusting the BS cut-off value to improve these aspects of the BS, with a particular emphasis on specificity and preventing overdiagnoses in those from more hypermobile populations [9, 10, 54, 56]. Indeed, the consensus of several researchers is that GJH should reflect an abnormality in the physiological context, and the categorisation of the BS values should, therefore, be more comparable to that of a reference range. Hence, it is suggested that the cut-off value is adjusted to allow identification of only those with a more extreme presentation within that population, i.e. a score that is 2 S.D. above the mean, or in the upper 5%, within each age, sex, and race category [10, 54, 55]. However, such a recommendation may not be appropriate for several reasons. First, the assertion that GJH is a physiological abnormality may not be medically accurate in this context. Here, the term ‘physiological abnormality’ is more appropriately applied to the joint that exceeds its normal ROM. The term GJH was then intended to describe the presentation of this physiological abnormality, i.e. JH, as systemic rather than localised, something this review has demonstrated may not be sufficiently reflected in the BS value itself, nor facilitated through adjustment to its cut-off point. Second, the higher prevalence observed in certain populations may not constitute instances of overdiagnoses, but may be a genuine finding of higher prevalence. For example, the increased prevalence of GJH seen in females is also associated with an increased symptomatic presentation and, therefore, likely to represent increased penetrance of an underlying HCTD [78]. If the cut-off value is raised further for specific populations to prevent ‘overdiagnosis’, those presenting with genuine GJH may be prevented from an accurate diagnosis without the additional hypermobile joints needed to meet the raised cut-off. Ethical concerns then also arise, if it appears a diagnosis is restricted to meet a specific prevalence rate, rather than reflecting the physiological presentation of a condition.

Additional joints could be included in the BS and a new cut-off point established to better aid a valid identification of GJH. Indeed, making use of the existing Hospital Del Mar or Rotès–Quérol scoring systems, which are effectively extensions of the BS could also be considered [6]. The validity and reliability of these scoring systems are difficult to determine since published findings into these aspects are scarce [6, 47, 48]. However, since such scoring systems better reflect the definition of GJH, they may be more suitable for diagnostics than the BS. Fundamentally, however, they still present with the same limitation. They do not directly measure the systemic nature of an individuals’ JH, and only count the number of select hypermobile joints an individual presents with.

Further suggestions to adjust the BS cut-off could be made for the purposes of better identifying those at risk of pathologic sequalae. Indeed, several studies have demonstrated that a higher BS is associated with greater pain persistence and recurrence [72, 73]. While this may be appropriate for preventative medicine, from a diagnostic perspective, it seems unnecessary to use predictive methods to categorize future symptomatology when a simple consultation with the patient can directly confirm the presence of symptoms. Consequently, raising the cut-off for this purpose or any of the aforementioned reasons would further diminish the sensitivity of the BS. This increases the risk of generating false negatives, and further excludes those with borderline GJH but a true systemic HCTD presentation from receiving an accurate diagnosis.

Indeed, this is an issue that has already been demonstrated and recently highlighted in a case study of two patients with the classical form of EDS (cEDS) [79]. An official diagnosis of cEDS is now only confirmed with molecular testing; however, cases are suspected and provisional diagnoses made based on clinical features, of which one of the major criteria is GJH with a BS of ≥ 5 [8]. The case study describes two patients, the index case and her mother, who showed subtle features of a connective tissue disorder with a suspicion of hEDS, yet did not fulfil the 2017 criteria for any form of EDS. Both patients scored ‘negatively’ on the BS, with scores of 3 and 4 out of 9, respectively; however, cEDS was suspected due to the characteristic presence of marked skin hyperextensibility in the mother. Subsequent molecular testing revealed a COL5A1 splice mutation in both patients confirming a diagnosis of cEDS. It is of note, however, that had the diagnosis, or indeed the decision to proceed to genetic testing remained dependent on the BS, these patients would not have received a diagnosis for any form of EDS [79]. This is particularly relevant for cases of hEDS, of which the molecular basis remains to be identified, and as such, the potential for misdiagnosis greater. It is clear that further research is needed to better understand how JH presents in the various forms of EDS and other related HCTDs, and the most effective method with which to clinically identify these presentations.

This narrative review has considered a large range of studies in the literature, which has allowed for a comprehensive discussion of the topic. This has highlighted several shortcomings in aspects of the BS’ validity and reliability, which has implications for its use as a diagnostic tool. However, the limitations of this review should also be recognised. Since studies adopt different versions of the BS, and interpretations of a GJH classification between them, this may have influenced their reported outcomes. Furthermore, since a formal meta-analysis was not performed, a formal evaluation of study methodology or a risk of bias assessment did not take place. As such, this review may have been influenced by studies of poor quality or ones containing bias. Despite this, the review has still demonstrated that insufficient evidence exists to justify use of the BS as a method to exclude the presence of GJH, or to differentiate GJH from localised hypermobility.

## Conclusion

The evidence presented here brings into question the validity of the BS as a direct and indirect indicator of GJH, and disputes its continued use as a diagnostic tool. Alternative assessment methods and tools exist, however, with over 300 joints in the body, it is unlikely any single assessment method will ever truly capture all variable presentations of GJH. Consequently, this has highlighted the desperate need for a clinically significant diagnostic marker(s) for the hypermobility disorders that would render such assessment methods redundant. Until such a time comes, use of the BS as a diagnostic tool should be one used with caution. Indeed, this review has demonstrated that a change in clinical thinking is required. In particular, the current use of a negative BS to exclude the presence of GJH is a practice that must be discontinued. Ultimately, this is not only diagnostically inaccurate, but could also deny a patient their fundamental right to a correct diagnosis, and hence access to appropriate support. Instead, the BS should be used as intended, i.e. as an initial screening method, after which other notable joints, for example, the shoulder, hips, ankles and remaining digits, could be examined until the clinician is satisfied that no evidence of systemic JH conclusively exists, nor any associated syndromic features, before excluding HCTDs as a diagnosis. Greater emphasis should now be placed on a clinician’s judgement to identify or exclude GJH according to its definition: the presence of JH at both the major and minor joints of the four limbs and axial skeleton.

## References

[1] Castori M, Tinkle B, Levy H, Grahame R, Malfait F, Hakim A (2017). A framework for the classification of joint hypermobility and related conditions. Am J Med Genet C Semin Med Genet.

[2] Grahame R (1999). Joint hypermobility and genetic collagen disorders: are they related?. Arch Dis Child.

[3] Demmler JC, Atkinson MD, Reinhold EJ, Choy E, Lyons RA, Brophy ST (2019). Diagnosed prevalence of Ehlers-Danlos syndrome and hypermobility spectrum disorder in Wales, UK: a national electronic cohort study and case–control comparison. BMJ Open.

[4] Carter C, Wilkinson J (1964). Persistent joint laxity and congenital dislocation of the hip. J Bone Joint Surg Br.

[5] Beighton P, Solomon L, Soskolne CL (1973). Articular mobility in an African population. Ann Rheum Dis.

[6] Bulbena A, Duró JC, Porta M, Faus S, Vallescar R, Martín-Santos R (1992). Clinical assessment of hypermobility of joints: assembling criteria. J Rheumatol.

[7] Terry RH, Palmer ST, Rimes KA, Clark CJ, Simmonds JV (2015). Horwood JP (2015) Living with joint hypermobility syndrome: patient experiences of diagnosis, referral and self-care. Fam Pract.

[8] Malfait F, Francomano C, Byers P, Belmont J, Berglund B, Black J, Bloom L, Bowen JM, Brady AF, Burrows NP, Castori M, Cohen H, Colombi M, Demirdas S, De Backer J, De Paepe A, Fournel-Gigleux S, Frank M, Ghali N, Giunta C, Grahame R, Hakim A, Jeunemaitre X, Johnson D, Juul-Kristensen B, Kapferer-Seebacher I, Kazkaz H, Kosho T, Lavallee ME, Levy H, Mendoza-Londono R, Pepin M, Pope FM, Reinstein E, Robert L, Rohrbach M, Sanders L, Sobey GJ, Van Damme T, Vandersteen A, van Mourik C, Voermans N, Wheeldon N, Zschocke J, Tinkle B (2017). The 2017 international classification of the Ehlers-Danlos syndromes. Am J Med Genet C Semin Med Genet.

[9] Smits-Engelsman B, Klerks M, Kirby A (2011). Beighton score: a valid measure for generalized hypermobility in children. J Pediatr.

[10] Singh H, McKay M, Baldwin J, Nicholson L, Chan C, Burns J, Hiller CE (2017). Beighton scores and cut-offs across the lifespan: cross-sectional study of an Australian population. Rheumatology (Oxford).

[11] Sauers EL, Borsa PA, Herling DE, Stanley RD (2001). Instrumented measurement of glenohumeral joint laxity and its relationship to passive range of motion and generalized joint laxity. Am J Sports Med.

[12] Whitehead NA, Mohammed KD, Fulcher ML (2018). Does the Beighton score correlate with specific measures of shoulder joint laxity?. Orthop J Sports Med.

[13] Ranalletta M, Bongiovanni S, Suarez F, Ovenza JM, Maignon G (2012). Do patients with traumatic recurrent anterior shoulder instability have generalized joint laxity?. Clin Orthop Relat Res.

[14] Johannessen EC, Reiten HS, Løvaas H, Maeland S, Juul-Kristensen B (2016). Shoulder function, pain and health related quality of life in adults with joint hypermobility syndrome/Ehlers-Danlos syndrome-hypermobility type. Disabil Rehabil.

[15] Morlino S, Dordoni C, Sperduti I, Venturini M, Celletti C, Camerota F, Colombi M, Castori M (2017). Refining patterns of joint hypermobility, habitus, and orthopedic traits in joint hypermobility syndrome and Ehlers-Danlos syndrome, hypermobility type. Am J Med Genet A.

[16] Rombaut L, Malfait F, Cools A, De Paepe A, Calders P (2010). Musculoskeletal complaints, physical activity and health-related quality of life among patients with the Ehlers-Danlos syndrome hypermobility type. Disabil Rehabil.

[17] Nourissat G, Vigan M, Hamonet C, Doursounian L, Deranlot J (2018). Diagnosis of Ehlers-Danlos syndrome after a first shoulder dislocation. J Shoulder Elbow Surg.

[18] Cypel D (2019). Gleno-humeral abduction measurement in patients with Ehlers-Danlos syndrome. Orthop Traumatol Surg Res.

[19] Pearsall AW, Kovaleski JE, Heitman RJ, Gurchiek LR, Hollis JM (2006). The relationships between instrumented measurements of ankle and knee ligamentous laxity and generalized joint laxity. J Sports Med Phys Fitness.

[20] Pietrzak K, Nowocień K, Kraszewski B (2020). Flexibility does not affect the dorsiflexion of foot and the popliteal angle in young adults. Foot Ankle Surg.

[21] Grip H, Hager C (2013). A new approach to measure functional stability of the knee based on changes in knee axis orientation. J Biomech.

[22] Benhamu-Benhamu S, Garcia-de-la-Pena R, Gijon-Nogueron G, Jimenez-Cristino MD, Gordillo-Fernandez LM, Dominguez-Maldonado G (2018). Range of Ankle Dorsiflexion in a Group of Adults with Ligamentous Laxity. J Am Podiatr Med Assoc.

[23] Lundberg G, Gerdle B (1999). The relationships between spinal sagittal configuration, joint mobility, general low back mobility and segmental mobility in female homecare personnel. Scand J Rehabil Med.

[24] Kim HJ, Yeom JS, Lee DB, Kang KT, Chang BS, Lee CK (2013). Association of benign joint hypermobility with spinal segmental motion and its clinical implication in active young males. Spine (Phila Pa 1976).

[25] Klemp P, Chalton D (1989). Articular mobility in ballet dancers. A follow-up study after four years. Am J Sports Med.

[26] Gajdosik RL, Albert CR, Mitman JJ (1994). Influence of hamstring length on the standing position and flexion range of motion of the pelvic angle, lumbar angle, and thoracic angle. J Orthop Sports Phys Ther.

[27] Hamonet C, Brock I (2015). Joint mobility and Ehlers-Danlos syndrome, (EDS) new data based on 232 cases. J Arthritis.

[28] Corten L, Ferguson G, Smits-Engelsman B (2020). Does the item ‘hands on floor’ add value to the Beighton score in identifying joint hypermobility?. Eur J Rheumatol.

[29] Czaprowski D, Kedra A, Pawlowska P, Kolwicz-Ganko A, Leszczewska J, Tyrakowski M (2015). The examination of the musculoskeletal system based only on the evaluation of pelvic-hip complex muscle and trunk flexibility may lead to failure to screen children for generalized joint hypermobility. PLoS ONE.

[30] Greenwood NL, Duffell LD, Alexander CM, McGregor AH (2011). Electromyographic activity of pelvic and lower limb muscles during postural tasks in people with benign joint hypermobility syndrome and non hypermobile people. A pilot study. Man Ther.

[31] Mitakides J, Tinkle BT (2017). Oral and mandibular manifestations in the Ehlers-Danlos syndromes. Am J Med Genet C Semin Med Genet.

[32] Conti PC, Miranda JE, Araujo CR (2000). Relationship between systemic joint laxity, TMJ hypertranslation, and intra-articular disorders. Cranio.

[33] Chiodelli L, Pacheco AB, Missau TS, Silva AM, Correa EC (2016). Influence of generalized joint hypermobility on temporomandibular joint and dental occlusion: a cross-sectional study. CoDAS.

[34] Wang HY, Shih TT, Wang JS, Shiau YY, Chen YJ (2012). Temporomandibular joint structural derangement and general joint hypermobility. J Oral Facial Pain Headache.

[35] Saez-Yuguero Mdel R, Linares-Tovar E, Calvo-Guirado JL, Bermejo-Fenoll A, Rodriguez-Lozano FJ (2009). Joint hypermobility and disk displacement confirmed by magnetic resonance imaging: a study of women with temporomandibular disorders. Oral Surg Oral Med Oral Pathol Oral Radiol Endod.

[36] Pasinato F, Souza JA, Correa EC, Silva AM (2011). Temporomandibular disorder and generalized joint hypermobility: application of diagnostic criteria. Braz J Otorhinolaryngol.

[37] Winocur E, Gavish A, Halachmi M, Bloom A, Gazit E (2000). Generalized joint laxity and its relation with oral habits and temporomandibular disorders in adolescent girls. J Oral Rehabil.

[38] Wolf JM, Schreier S, Tomsick S, Williams A, Petersen B (2011). Radiographic laxity of the trapeziometacarpal joint is correlated with generalized joint hypermobility. J Hand Surg Am.

[39] van Andel CJ, Roescher WB, Tromp MF, Ritt MJ, Strackee SD, Veeger DH (2008). Quantification of wrist joint laxity. J Hand Surg Am.

[40] Al-Rawi ZS, Al-Aszawi AJ, Al-Chalabi T (1985). Joint mobility among university students in Iraq. Br J Rheumatol.

[41] Rikken-Bultman DG, Wellink L, van Dongen PW (1997). Hypermobility in two Dutch school populations. Eur J Obstet Gynecol Reprod Biol.

[42] Zakas A, Vergou A, Grammatikopoulou MG, Zakas N, Sentelidis T, Vamvakoudis S (2003). The effect of stretching during warming-up on the flexibility of junior handball players. J Sports Med Phys Fitness.

[43] Petrofsky JS, Laymon M, Lee H (2013). Effect of heat and cold on tendon flexibility and force to flex the human knee. Med Sci Monit.

[44] Brodowicz GR, Welsh R, Wallis J (1996). Comparison of Stretching with Ice, Stretching with Heat, or Stretching Alone on Hamstring Flexibility. J Athl Train.

[45] Zazulak BT, Paterno M, Myer GD, Romani WA, Hewett TE (2006). The effects of the menstrual cycle on anterior knee laxity: a systematic review. Sports Med.

[46] McHugh ML (2012). Interrater reliability: the kappa statistic. Biochem Med (Zagreb).

[47] Schlager A, Ahlqvist K, Rasmussen-Barr E, Bjelland EK, Pingel R, Olsson C, Nilsson-Wikmar L, Kristiansson P (2018). Inter- and intra-rater reliability for measurement of range of motion in joints included in three hypermobility assessment methods. BMC Musculoskelet Disord.

[48] Juul-Kristensen B, Rogind H, Jensen DV, Remvig L (2007). Inter-examiner reproducibility of tests and criteria for generalized joint hypermobility and benign joint hypermobility syndrome. Rheumatology (Oxford).

[49] Junge T, Jespersen E, Wedderkopp N, Juul-Kristensen B (2013). Inter-tester reproducibility and inter-method agreement of two variations of the Beighton test for determining Generalised Joint Hypermobility in primary school children. BMC Pediatr.

[50] Hansen A, Damsgaard R, Kristensen JH, Bagger J, Remvig L (2002). Interexaminer reliability of selected tests for hypermobility. J Orth Med.

[51] Boyle KL, Witt P, Riegger-Krugh C (2003). Intrarater and interrater reliability of the beighton and horan joint mobility index. J Athl Train.

[52] Hirsch C, Hirsch M, John MT, Bock JJ (2007). Reliability of the Beighton Hypermobility Index to determinate the general joint laxity performed by dentists. J Orofac Orthop.

[53] Juul-Kristensen B, Schmedling K, Rombaut L, Lund H, Engelbert RHH (2017). Measurement properties of clinical assessment methods for classifying generalized joint hypermobility-A systematic review. Am J Med Genet C Semin Med Genet.

[54] Jansson A, Saartok T, Werner S, Renström P (2004). General joint laxity in 1845 Swedish school children of different ages: age- and gender-specific distributions. Acta Paediatr.

[55] Fairbank JC, Pynsent PB, Phillips H (1984). Quantitative measurements of joint mobility in adolescents. Ann Rheum Dis.

[56] Clinch J, Deere K, Sayers A, Palmer S, Riddoch C, Tobias JH, Clark EM (2011). Epidemiology of generalized joint laxity (hypermobility) in fourteen-year-old children from the UK: a population-based evaluation. Arthritis Rheum.

[57] Larsson LG, Baum J, Mudholkar GS, Srivastava DK (1993). Hypermobility: prevalence and features in a Swedish population. Br J Rheumatol.

[58] Klemp P, Williams SM, Stansfield SA (2002). Articular mobility in Maori and European New Zealanders. Rheumatology (Oxford).

[59] Remvig L, Jensen DV, Ward RC (2007). Epidemiology of general joint hypermobility and basis for the proposed criteria for benign joint hypermobility syndrome: review of the literature. J Rheumatol.

[60] Wordsworth P, Ogilvie D, Smith R, Sykes B (1987). Joint mobility with particular reference to racial variation and inherited connective tissue disorders. Br J Rheumatol.

[61] Kwon J-W, Lee W-J, Park S-B, Kim MJ, Jang SH, Choi CK (2013). Generalized joint hypermobility in healthy female koreans: prevalence and age-related differences. Ann Rehabil Med.

[62] Birrell FN, Adebajo AO, Hazleman BL, Silman AJ (1994). High prevalence of joint laxity in West Africans. Br J Rheumatol.

[63] Abujam B, Aggarwal A (2014). Hypermobility is related with musculoskeletal pain in Indian school-children. Clin Exp Rheumatol.

[64] Gyldenkerne B, Iversen K, Roegind H, Fastrup D, Hall K, Remvig L (2007). Prevalence of general hypermobility in 12–13-year-old school children and impact of an intervention against injury and pain incidence. Adv Physiother.

[65] Lamari NM, Chueire AG, Cordeiro JA (2005). Analysis of joint mobility patterns among preschool children. Sao Paulo Med J.

[66] Qvindesland A, Jónsson H (1999). Articular hypermobility in Icelandic 12-year-olds. Rheumatology (Oxford).

[67] Russek LN, Errico DM (2016). Prevalence, injury rate and symptom frequency in generalized joint laxity and joint hypermobility syndrome in a "healthy" college population. Clin Rheumatol.

[68] Seçkin U, Tur BS, Yilmaz O, Yagci I, Bodur H, Arasil T (2005). The prevalence of joint hypermobility among high school students. Rheumatol Int.

[69] Quatman CE, Ford KR, Myer GD, Paterno MV, Hewett TE (2008). The effects of gender and pubertal status on generalized joint laxity in young athletes. J Sci Med Sport.

[70] Remvig L, Kümmel C, Kristensen JH, Boas G, Juul-Kristensen B (2011). Prevalence of generalized joint hypermobility, arthralgia and motor competence in 10-year-old school children. Int Musculoskelet Med.

[71] Mikkelsson M, Salminen JJ, Kautiainen H (1996). Joint hypermobility is not a contributing factor to musculoskeletal pain in pre-adolescents. J Rheumatol.

[72] El-Metwally A, Salminen JJ, Auvinen A, Kautiainen H, Mikkelsson M (2004). Prognosis of non-specific musculoskeletal pain in preadolescents: a prospective 4-year follow-up study till adolescence. Pain.

[73] El-Metwally A, Salminen JJ, Auvinen A, Kautiainen H, Mikkelsson M (2005). Lower limb pain in a preadolescent population: prognosis and risk factors for chronicity–a prospective 1- and 4-year follow-up study. Pediatrics.

[74] Engelbert RHH, Bank RA, Sakkers RJB, Helders PJM, Beemer FA, Uiterwaal CSPM (2003). Pediatric generalized joint hypermobility with and without musculoskeletal complaints: a localized or systemic disorder?. Pediatrics.

[75] Castori M, Camerota F, Celletti C, Danese C, Santilli V, Saraceni VM, Grammatico P (2010). Natural history and manifestations of the hypermobility type Ehlers-Danlos syndrome: a pilot study on 21 patients. Am J Med Genet A.

[76] Castori M, Sperduti I, Celletti C, Camerota F, Grammatico P (2011). Symptom and joint mobility progression in the joint hypermobility syndrome (Ehlers-Danlos syndrome, hypermobility type). Clin Exp Rheumatol.

[77] Castori M, Dordoni C, Morlino S, Sperduti I, Ritelli M, Valiante M, Chiarelli N, Zanca A, Celletti C, Venturini M, Camerota F, Calzavara-Pinton P, Grammatico P, Colombi M (2015). Spectrum of mucocutaneous manifestations in 277 patients with joint hypermobility syndrome/Ehlers-Danlos syndrome, hypermobility type. Am J Med Genet C Semin Med Genet.

[78] Castori M, Camerota F, Celletti C, Grammatico P, Padua L (2010). Ehlers-Danlos syndrome hypermobility type and the excess of affected females: possible mechanisms and perspectives. Am J Med Genet A.

[79] Colombi M, Dordoni C, Cinquina V, Venturini M, Ritelli M (2018). A classical Ehlers-Danlos syndrome family with incomplete presentation diagnosed by molecular testing. Eur J Med Genet.

